# Natural Products and Derivatives Targeting Metabolic Reprogramming in Colorectal Cancer: A Comprehensive Review

**DOI:** 10.3390/metabo14090490

**Published:** 2024-09-09

**Authors:** Mengyu Wang, Liqun Qu, Xinying Du, Peng Song, Jerome P. L. Ng, Vincent Kam Wai Wong, Betty Yuen Kwan Law, Xianjun Fu

**Affiliations:** 1 Nehr’s Biophysics Laboratory for Innovative Drug Discovery, State Key Laboratory of Quality Research in Chinese Medicine, Macau University of Science and Technology, Macau 999078, China; 2109853dcw30003@student.must.edu.mo (M.W.);; 2 Research Institute for Marine Traditional Chinese Medicine, Key Laboratory of Marine Traditional Chinese Medicine in Shandong Universities, Shandong Engineering and Technology Research Center on Omics of Traditional Chinese Medicine, Shandong University of Traditional Chinese Medicine, Jinan 250355, China; 3 Qingdao Academy of Chinese Medical Sciences Shandong University of Traditional Chinese Medicine, Qingdao Key Laboratory of Research in Marine Traditional Chinese Medicine, Qingdao Key Technology Innovation Center of Marine Traditional Chinese Medicine’s Deep Development and Industrialization, Qingdao 266114, China

**Keywords:** colorectal cancer, metabolic reprogramming, natural products, glycolysis, oxidative phosphorylation, metabolic enzymes

## Abstract

Metabolic reprogramming is a critical pathogenesis of colorectal cancer (CRC), referring to metabolic disorders that cancer cells make in response to the stimulating pressure. Metabolic reprogramming induces changes in genetic material and promotes CRC progression and has been proven to be an efficient target of CRC. As natural products have garnered interest due to notable pharmacological effects and potential in counteracting chemoresistance, an increasing body of research is delving into the impact of these natural products on the metabolic reprogramming associated with CRC. In this review, we collected published data from the Web of Science and PubMed, covering the period from January 1980 to October 2023. This article focuses on five central facets of metabolic alterations in cancer cells, glucose metabolism, mitochondrial oxidative phosphorylation (OXPHOS), amino acid metabolism, fatty acid synthesis, and nucleotide metabolism, to provide an overview of recent advancements in natural product interventions targeting metabolic reprogramming in CRC. Our analysis underscores the potential of natural products in disrupting the metabolic pathways of CRC, suggesting promising therapeutic targets for CRC and expanding treatment options for metabolic-associated ailments.

## 1. Introduction

Colorectal cancer (CRC) is the third most common cancer in the world, which accounts for approximately 10% of all cancer cases [1]. As the second leading cause of cancer-related mortality worldwide, CRC seriously threatens people’s health and places an enormous economic burden on the world [2]. With the introduction and in-depth study of the tumor metabolism hypothesis that tumor cells preferentially utilize glycolysis even in the presence of oxygen, known as the Warburg effect, metabolic dysregulation is recognized as one of the fundamental hallmarks of cancer, in alignment with genomic instability, tumor immune evasion, and the tumor microenvironment [3,4]. Tumor cells experience various metabolic alterations to meet their energy needs for rapid growth and multiplication, thereby adjusting to the tumor microenvironment, like hypoxia [5,6]. In contrast to the main energy supply mode of normal cells—mitochondrial respiration and OXPHOS—the metabolic pattern of tumor cells is significantly regulated, including but not limited to aerobic glycolysis, increased de novo biosynthesis of fatty acids (FA), massive consumption of glutamine and hypermetabolism of nucleotides [7]. Targeting the metabolic heterogeneity of CRC cells is a promising therapeutic approach. 5-Fluorouracil (5-FU) targets nucleotide metabolism thymidylate synthase (TS) with excellent clinical results of CRC [8]. Although various metabolic enzymes inhibitors are currently under development, issues such as unstable efficacy and adverse events have limited their progress [9]. Therefore, the search for safe and effective alternative drugs has become imperative.

Natural products not only possess various advantages, such as abundant sources, multiple bioactivities, and diverse varieties, but also have demonstrated potential in CRC therapeutics [10,11]. In addition, natural products can regulate metabolism in various diseases such as obesity and diabetes [12,13,14]. Therefore, natural products serve as a valuable reservoir of candidate compounds for developing targeted metabolic enzyme inhibitors. Advancing research continues to substantiate the role of these natural products in remodeling the metabolic landscape of CRC cells, influencing both the metabolic flux and the enzymatic activities which are pivotal to the neoplastic phenotype, thereby refining their relevance in antitumor strategies [15,16].Therefore, this review outlines principal metabolic enzymes regulated in CRC and provides an overview of the impact of natural compounds on these metabolic enzymes, associated proteins, oncogenes, and intertwined pathways specific to CRC. In summary, we hope this article gives a reference for the screening of antitumor natural compounds with significant antimetabolic effects

## 2. Natural Products Regulate Multiple Metabolisms in CRC

**Figure 1:**
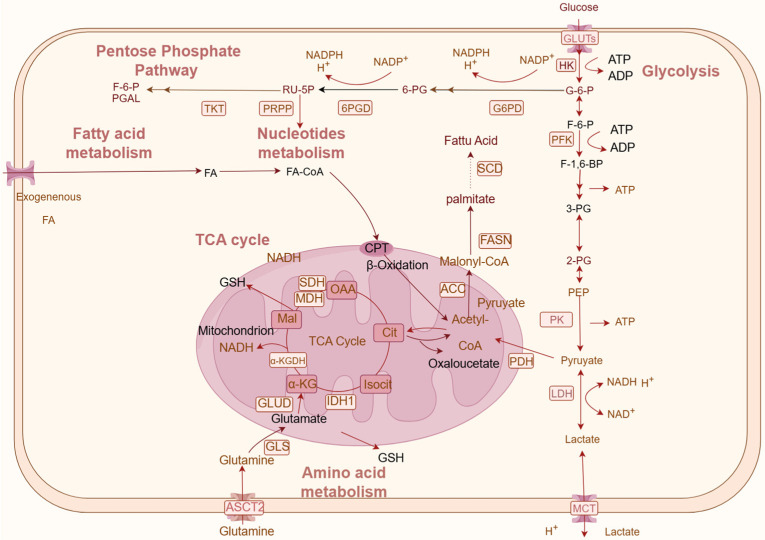
**Schematic of natural products regulating key metabolism factors and metabolism pathways in CRC.** Metabolic pathways mainly consist of glucose metabolism (glycolysis, the PPP pathway, and the TCA cycle), lipid metabolism, amino acid metabolism, and nucleotide metabolism. Rectangular boxes indicate metabolic enzymes; arrows represent metabolic directions. OAA: oxaloacetate; Cit: citric acid; isocit: citric acid; α-KG: alpha-ketoglutarate; Mal: malic acid; 3-PG: 3-phosphoglyceric acid; RU-5P: ribulose 5-phosphate; 6-PG: 6-phosphogluconic acid (created on www.figdraw.com).

Natural products regulate the synthesis and catabolism of diverse substances, such as glucose, amino acids, lipids, and nucleotides, thereby reducing CRC proliferation and invasion. The metabolic pathways include various metabolic enzymes, of which activities regulate substance metabolism in cells and determine the rate and direction of the entire metabolic pathway. This section seeks to encapsulate the impact of natural products on metabolic enzymes and metabolic products (Figure 1).

### 2.1. Natural Products Regulate Glucose Metabolism

In CRC, abnormal glucose metabolism is a common alteration, characterized by a preference for increased glycolysis for energy production, which disrupts the balance of the tricarboxylic acid (TCA) cycle [17,18]. Furthermore, the pentose phosphate pathway (PPP), which supplies ribonucleotide and Nicotinamide Adenine Dinucleotide Phosphate (NADPH) as required, was also enhanced in CRC cells [19]. Such a metabolic reconfiguration not only satiates the energy demands of proliferating cancer cells but also provides essential macromolecules including lipids, amino acids, nucleic acids, and abundant biosynthetic intermediates for biosynthesis [20]. Lactate, another metabolite of glycolysis, is excreted to the extracellular matrix by MCT. The extracellular accumulation of lactate results in the tumor microenvironment being characterized by hypoxia and acidity, propelling tumor growth, invasion, and metastasis [21].

The control of glucose metabolism is contingent on the activities of enzymes critical to glucose metabolism, including key glucose transporter (GLUT) and monocarboxylate transporter (MCTS) glycolysis enzymes, such as phosphofructokinase (PFK), hexokinase (HK), lactate dehydrogenase (LDHA), pyruvate kinase 1/2 (PKM1/2), and isocitrate dehydrogenase (IDH) (Figure 1). Inhibition of glycolytic enzyme activity can reduce glycolytic flux, diminish glucose uptake, and lactate production in CRC cells, thereby slowing CRC progression (Table 1).

#### 2.1.1. Natural Products Regulate Glycolysis

One molecule of glucose is metabolized into two molecules of pyruvate in glycolysis, serving as the common initial step for both anaerobic and aerobic glucose oxidation [22]. HK is a key enzyme that catalyzes the first rate-limiting step in the phosphorylation of glucose to generate glucose-6-phosphate (G-6-P). There are five distinct isoforms within the mammalian HK family: HKI, HKII, HKIII, HKIV, and HK domain-containing protein 1 (HKDC1). Among them, the isoenzyme HK2 is most relevant for energy metabolism in tumor cells, which is closely associated with the initiation and progression of tumorigenesis [23,24,25]. PFK1 acts as a critical glycolytic agent regulating the second rate-limiting step of glycolysis, transforming fructose-6-phosphate (F-6-P) into fructose-1, 6-bisphosphate (F-1,6-BP) [26]. PK is a key controller in the process of glycolysis, and catalyzes the formation of pyruvate from the substrate, among which the M2 subtype PKM2 confers a growth advantage to tumor cells, allowing them to prosper and adjust in their surrounding environment [27].

The GLUT is the critical rate-determining enzyme in glucose metabolism, orchestrating the transmembrane transport of glucose [28]. Thirteen distinct GLUT isoforms have been identified. Structural variances among the GLUT isoforms give rise to distinct functional attributes and distribution patterns [29]. Notably, GLUT1-5 have been extensively characterized in terms of their structural and functional nuances. GLUT1 and GLUT3, which exhibit a heightened affinity for glucose, serve as the foundational transporters mediating cellular glucose uptake [30,31]. These transporters are ubiquitously expressed throughout the organism and exhibit a profound association with neoplastic cells.

LDHA plays a crucial role as an active enzyme in the glycolytic cascade, catalyzing the conversion of pyruvate to lactate under anaerobic conditions [32]. Lactate not only emerges as an anaerobic byproduct but also acts as a catalyst within the tumor microenvironment, where an acidic milieu confers a proliferative advantage to neoplastic cells [33]. The MCT represents a significant molecular entity crucial for the maintenance of glycolytic metabolism, exhibiting dual functionality as both a lactate transporter and a pH regulator [34]. The MCT family encompasses a total of 14 members, with the initial four (MCT1-4) identified as facilitators of proton-coupled transport of monocarboxylic acids across the plasma membrane [35]. Inhibition of MCT disrupts cellular and extracellular balance; that is, it affects pH homeostasis, induces apoptosis, and reduces tumor angiogenesis, invasion, and metastasis [36].

Wogonin, a flavonoid isolated from *Scutellaria baicalensis* Georgi., has shown potent antitumor effects [37]. Wogonin downregulates the expression of GLUT1 and the activities of key glycolytic enzymes (PGM, HK2, GLUT1, and LDHA) and PDHK1 [38]. Although PDHK itself is not involved in the chemistry of glycolysis, it is linked to glycolysis because it regulates the subsequent metabolic pathway of pyruvate produced from glycolysis. PDHK acts as a regulator, influencing the fate of pyruvate after glycolysis, rather than being directly involved in its breakdown [39]. Saponin monomer 13 of the dwarf lilyturf tuber (DT-13) is the major steroidal saponin in *Liriope muscari* (Decne). DT-13 significantly downregulates GLUT1 to inhibit glycolysis and alter energy homeostasis in cancer cells [15]. DT13 showed better activity against two CRC cells (SW480, COLO205) compared to 5-FU (Table 1). Diosgenin (DSG) mediates GLUT proteins (GLUT3 and GLUT4) to reduce aerobic glycolysis, and downregulates pyruvate carboxylase (PC), increasing the proximity of CRC cells to hypoxic glycolysis and OXPHOS [40]. DSG exhibits better drug toxicity against SW1116 cells compared to 5-FU, while demonstrating weaker toxicity towards normal cells (Table 1). The apple polyphenol phloretin (Ph) inhibits tumor cell invasion by inhibiting the activity of GLUT2 and inducing HNF6-mediated p53 activation [41]. *Sophora flavescens* Aiton(also known as “Ku Shen”) is a Chinese traditional medicinal herb, with its cold nature and bitter taste, and traditionally efficacy are heat-clearing and dampness-drying. It can be utilized for the treatment of symptoms associated with CRC, including rectal bleeding [42]. Matrine, an alkaloid extracted from the root of Kushen, exhibits outstanding pharmacological activity, including cardiovascular and cerebrovascular protection, anticancer, and anti-inflammatory activity [43,44,45,46]. Matrine reversed the Warburg effect by downregulating Hypoxia-inducible factor 1-alpha (HIF-1α) and inhibiting the expression of its downstream target (GLUT1, HK2, and LDHA) [47]. Additionally, it demonstrates excellent anti-CRC activity comparable to that of 5-FU (Table 1). Oxymatrine inhibits cell metastasis possibly by blocking aerobic glycolysis, which depends on dual inhibition of PKM2 and downregulation of GLUT1 expression [48].

Parthenolide (PT), an efficacious compound isolated from medicinal plants, has profound anti-inflammatory properties [49]. Derivatives of PT impeded the nuclear translocation of PKM2, inducing a change in metabolic pattern from aerobic glycolysis to oxidative phosphorylation, and thereby exerting antiproliferative effects on HT29 and SW480 cells [50]. PT exhibits lower IC50 values compared to 5-FU in HCT116, HT29, and SW480 cell lines, as well as in normal cells. This suggests high cytotoxicity and potential off-target effects. Kaempferol (KP) is a flavonol that is extracted from many medicinal plants. It is recognized for diversiform and outstanding pharmacological activities [51,52]. Kaempferol promotes the expression of miR-339-5p, which in turn, directly targets hnRNPA1 and PTBP1, diminishing the expression of PKM2 and inducing the expression of PKM1, thus inhibiting glycolysis and the growth of colorectal cancer [53].

Tannic acid (TA), is a natural polyphenolic acid found predominantly in grapes and green tea, that possesses potent antioxidant and anticancer properties [54]. TA inhibits CRC cell proliferation by binding to lysine 433, triggering PKM2 tetramer dissociation and blocking PKM2 metabolic activity [55]. Berberine, an isoquinoline alkaloid extracted from the herb *Coptis chinensis* Franch., displays antiproliferative and anticancer effects [56]. Berberine suppress the enzymatic activity of PKM2 and downregulated glycolysis [57]. Among other plants, apigenin (AP), which was identified in celery, inhibits glycolysis in cancer cells by inhibiting the activity and expression of PKM2. It reduces the PKM2/PKM1 ratio in HCT116 cells by interrupting the β-catenin/c-Myc/PTBP1 signaling pathway [58]. Resveratrol is a naturally occurring antimicrobial polyphenol (phytoalexin) present in a variety of plants. The principal sources of natural resveratrol include grapes, especially specific cultivars such as *Vitis amurensis* and certain Rootstock varieties, as well as the medicinal plant *Polygonum cuspidatum* [59,60,61]. Resveratrol downregulates PKM2 expression by upregulating micro RNA326 (miR-326), thereby exerting antitumor effects through the induction of endoplasmic reticulum stress and mitochondrial fission [62]. Additionally, Resveratrol decreases glycolytic activity by inhibiting the activity of pyruvate kinase (PK) and LDH in Caco2 cells [63]. IC50 of berberine, AP and resveratrol are higher than 5FU, suggesting the capacity of these compounds to inhibit the proliferation of CRC cells may not related to cell toxicity, but regulation of metabolism.

Dioscin is a natural steroidal saponin extracted from a variety of herbs. Dioscin inhibits glycolysis by inducing Skp2 ubiquitination and inhibiting HK2 in tumor tissues, which is dependent on the Skp2-Akt-HK2 axis [64]. Wu found that dioscin promotes the interaction of c-Myc and FBW7 to promote c-myc ubiquitination, which in turn inhibits the expression of HK2 to reduce tumor glycolysis and induce cell apoptosis [65]. Curcumin inhibits the glycolytic activity of tumor cells by inhibiting the expression and activity of HK2 in HCT116 and HT29 cells, and induces the dissociation of mitochondrial HK2 through AKT phosphorylation, leading to mitochondria-mediated apoptosis [24]. Xanthohumol, a polyphenol chalcone from *Humulus lupulus* L., has been proven to have good antitumor activity [66]. Xanthohumol inhibits glycolytic activity by mediating Akt and promotes apoptosis through the downregulation of HK2 expression [67]. Thymoquinone, an important bioactive phytochemical component of *Nigella sativa* L., has antioxidation, antimicrobial, anti-inflammatory, metabolic syndrome, and other effects [68,69,70]. It inhibits the rate-limiting HK2 of glycolysis by regulating the PI3K/AKT axis [71], and shows greater cell toxicity to SW480 cell.

Oridonin, a diterpenoid extracted from *Isodon rubescens* (Hemsl.), which exhibits excellent activity of suppress multiple CRC cells (Table 1), regulates AMPK-related GLUT1 and MCT1 to affect glucose supply and lactate production, thereby affecting glucose metabolism and inducing autophagy through metabolic pathways [72]. Triterpenoids (TER) of *Rhus chinensis* Mill., with unsignificant cell toxicity (Table 1), dose-dependently inhibited the expression of the glucose metabolism enzymes GLUT1, LDHA, PKM2, and MCT1, which mediate lactate transport, and affect the growth and proliferation of tumor cells by affecting the ASIC2-mediated calcineurin/NFAT1 pathway [73]. Lycorine is a crystalline alkaloid extracted from the *Lycoris radiata* Herb. that has multiple pharmacological effects, including anti-inflammatory, antifungal, antiviral, and antitumor activities [74,75,76]. Lycorine interferes with the interaction between IDH1 and the deacetylase SIRT1, thereby significantly promoting the acetylation of IDH1 and driving the oxidative stress-dependent imbalance of mitochondrial dynamics to exert an anti-CRC effect [77]. Xanthatin is a bioactive sesquiterpene lactone isolated from *Xanthium strumarium* L. The inhibition of glycolysis by xanthatin may be related to the reduction in GLUT1 and MCT4 mRNA and protein levels. It promotes complex II activity and OXPHOS, leading to mitochondrial damage and resulting in cell death. Xanthatin inhibited the phosphorylation of mTOR, 4E-binding protein 1 (4E-BP1), and c-myc in HT-29 cells [78]. β-Caryophyllene (BCP) is a natural sesquiterpene extracted from various plants, such as cloves and cinnamon. BCP inhibits ART1-induced glycolysis and increases ATP and lactate production through the AKT/mTOR pathway, thereby increasing the expression of PKD1 and LDHA and affecting the proliferation and apoptosis of CT26 cells under high-glucose conditions [79]. Water extracts from the seeds of *Myristica fragrans* Houtt. (MF) inhibits the Warburg effect and cell growth by downregulating LDH enzyme activity in HT29 cells [80].

#### 2.1.2. Natural Products Regulate the TCA Cycle and Oxidative Phosphorylation

The TCA cycle represents the terminal metabolic pathway for the three principal nutrients—carbohydrates, lipids, and amino acids—and serves as the central hub interlinking the metabolism of these macromolecules. The TCA cycle is instrumental in generating metabolic intermediates and NADH to support cellular proliferation [81]. Pyruvate produced from glycolysis is converted into acetyl-CoA by the catalytic action of the pyruvate dehydrogenase (PDH) complex, after which it enters the TCA cycle. Except for the reactions catalyzed by citrate synthase and the oxoglutarate dehydrogenase (OGDH) complex, the majority of reactions within the cycle are reversible, with most intermediates capable of anaplerotic reactions. Fluxes through the TCA cycle are of paramount importance in metabolism and are frequently dysregulated in cancers, including CRC [82]. The investigation into enhancing pyruvate-mediated mitochondrial oxidation, modulating TCA cycle enzymes, and perturbing respiratory chain complexes has identified potential therapeutic targets [83]. The pyruvate dehydrogenase complex (PDC) is crucial for converting pyruvate to acetyl-CoA, a key substrate for the TCA cycle, de novo lipogenesis, and protein acetylation [84]. Notably, this complex is subject to negative regulation by pyruvate dehydrogenase kinases (PDKs), which are frequently overexpressed in cancer cells [85].

Following resveratrol intervention, a decrease in PDH activity has been observed in Caco-2 and HCT116 colorectal cancer cell lines, indicating that resveratrol-induced glycolytic reprogramming may occur through the modulation of PDP1 gene expression [86]. Withania somnifera is a traditional medicinal plant, of which main active compounds include Withanolides (Withaferin A, Withanolide A, Withanone), sitoindosides, Withanosides, and other alkaloids [87], and they have significant anticancer activity, antimicrobial, anti-inflammatory, and cardioprotective effects [88,89,90,91]. Alcoholic extract of withania somnifera increased the activities of key TCA cycle enzymes, including isocitrate dehydrogenase (ICDH), succinatede hydrogenase (SDH), malate dehydrogenase (MDH), Alpha-ketoglutarate dehydrogenase complex (α-KGDH) and four complex electron transport chain enzymes to normalize the levels of these enzymes in azoxymethane-induced experimental mice [92]. Pyruvate dehydrogenase kinase 1(PDK1) phosphorylates the PDH complex to inactive it, which is the critical point connecting aerobic glycolysis and the diaminobutoxy and TCA cycle (Figure 1). Hemistepsin A (HsA) is a sesquiterpene lactone isolated from the medical herb *Hemisteptia lyrata* (Bunge) Fisch. & C.A.Mey. HsA exhibits significant cytotoxicity against CRC cells while demonstrating relatively low toxicity towards normal cells (Table 1). HsA inhibits the activity of PDK1 by interfering with the interaction between PDK1 and the L2 lipoamide domain of PDH-E2, thus switching the metabolic pattern from glycolysis to OXPHOS. Therefore, the growth of CRC cells is suppressed, and mitochondrial ROS-mediated apoptosis is induced [93]. Diaminobutoxy-substituted isoflavonoid (DBI-1), a complex I inhibitor, has good synergy with the GLUT1 inhibitor BAY-876 to inhibit the survival of CRC cells both in vitro and in vivo [94]. DBT-1 exhibits superior inhibitory effects on CRC cell proliferation compared to 5-FU.

#### 2.1.3. Natural Products Regulate Pentose Phosphate Pathway

In addition to the aerobic and anaerobic decomposition of glucose, PPP also occurs in tissues involved in active lipid synthesis and proliferation (such as liver, adipose tissue, bone marrow, and tumor tissues), and is an important pathway for glucose oxidative decomposition [95]. The PPP starts from the intermediate product of glycolysis, G-6-P, to bypass G-6-P through two stages—oxidation and group transfer—ultimately producing F-6-P and glyceraldehyde 3-phosphate, thus returning to glycolysis. The PPP does not produce ATP, but it produces the phosphonic ribose and NADPH, which are essential for nucleic acid biosynthesis and various metabolic pathways. This pathway provides a carbon source and a hydrogen donor for a multitude of anabolic reactions within the body. [96]. In addition, NAPDH is not only involved in lipid cholesterol amino acid metabolism, but also an essential substance for maintaining the glutathione reduction state, which is highly important for ROS clearance [97]. Thus, the PPP is necessary to promote the growth of CRC cell growth and to produce NADPH to overcome oxidative stress.

6-phosphogluconate dehydrogenase (G6PD), 6-phosphogluconate lactonase (PGLS), and 6-phosphogluconate dehydrogenase (6PGD) are key enzymes involved in the oxidation of the PPP (Figure 1). Transketolase (TKT) and transaldolase (TAL) are the key enzymes involved in radical metastasis, and are closely related to tumor growth [98]. Sánchez-Tena demonstrated that the catechin derivative epicatechin gallate (ECG) significantly decreased glucose uptake and glutamate accumulation while concurrently augmenting glutamine consumption. The activity of critical enzymes associated with the PPP, namely, TKT and G6PD, was found to be inhibited [99]. At a concentration of 50 μM, resveratrol targeted the PPP and the talin–pFAK axis, effectively inhibiting CRC cell proliferation and inducing apoptosis. This inhibitory effect was concomitant with a decrease in the activity and/or protein expression levels of G6PD and TKT, culminating in the suppression of the PPP. Moreover, the impact of resveratrol was notably associated with alterations in both glycolytic and PPP activities [100]. Halofuginone (HF), a febrifugine derivative sourced from the traditional medicinal plant *Dichroa febrifuga* Lour., which significantly inhibited the viability of CRC cells, was observed to inactivate G6PD within the PPP posttreatment. This led to a reduction in NADPH levels, a decrease in glycolytic flux, and a decrease in the rate of glucose-derived TCA flux. Furthermore, CRC cells treated with HF exhibited downregulation of GLUT1 and HK2, ultimately resulting in the inhibition of cell proliferation and the induction of reactive oxygen species production [101].

**Table 1:** Natural products regulate glucose metabolism enzymes in CRC.

Chemical Class	Bioactive Compounds	Medicinal Plant	Cancer Model	IC50	Metabolic Regulation	Targets	Potential Mechanisms	References
Flavonoids	Wogonin	*Scutellaria baicalensis* Georgi.	HT-29, HCT116	-	Glycolysis	GLUT1, PGM, HK2, PDHK1, LDHA	Increased the expression of p53 and downregulates key glycolytic enzymes to inhibit colorectal cancer glycolysis	[38]
Flavonoids	Apple Polyphenol Phloretin	*Malus pumila* Mill.	HT29, COLO205, xenograft mouse model	-	Glucose metabolism	GLUT2	Inhibited GLUT2 protein expression and induces Hepatocyte nuclear factor 6 (HNF6) to activate GLUT2 and p53	[41]
Flavonoids	Silybin	*Silybum marianum*(L.) Gaertn.	LoVo WT LoVo DOX	-	Glucose metabolism	GLUT1	Targeted GLUTs to increase doxorubicin sensitivity and elude drug resistance	[102]
Flavonoids	Kaempferol	*Kaempferia galanga* L.	HCT116, DLD1	HCT116: 63.0 ± 12.9 μM DLD1: 98.3 ± 15.9 μM	Glycolysis	PKM2, hnRNPA1	Inhibited glycolysis and colon cancer growth by modulating miR-339-5p-hnRNPA1/PTBP1-PKM2 axis	[53]
Flavonoids	Apigenin	*Thymus mongolicus* Ronniger	HCT116, HT29, DLD1	HCT116: 27.9 ± 2.45 μM HT29: 48.2 ± 3.01 μM DLD1: 89.5 ± 4.89 μM	Glycolysis	PKM2	Bonded to PKM2 and inhibited PKM2 activity, and reduced PKM2/PKM1 by blocking β-catenin/c-Myc/PTBP1 signaling pathway	[58]
Flavonoids	Xanthohumol	*Humulus lupulus* L.	HCT116, HT29, SW620, SW480, LOVO, Xenograft mouse model		Glycolysis	HK2	Reduced Akt activity, and inhibited HK2 expression and glycolysis	[67]
Flavonoids	Quercetin	*Crataegus pinnatifida* Bge.	HCT15, RKO	HCT15:142.7 μM RKO:121.9 μM NCM460: >200 μM	Glycolysis	MCT1, MCT4	Decreased glucose consumption and lactate acid generation by inhibiting MCT, and enhanced the cytotoxicity of 5-FU	[103]
Isoflavonoid	Diaminobutoxy-substituted Isoflavonoid (DBI-1)	semisynthetic derivatives	LS174T Pt2377 Xenograft mouse model	LS174T: 1.2 μM Pt2377: 1.4 μM	TCA cycle	GLUT1 mitochondrial complex I	Inhibits mitochondrial complex I, and combined with GLUT1 inhibitor, BAY-876, synergistically inhibited colorectal cancer	[94]
Steroidal saponins	Saponin monomer 13 of the dwarf lilyturf tuber (DT13)	*Liriope muscari* (Decne.)	HCT15,HT29, COLO205, HCT116, SW480,SW620 Orthotopic implantation mouse model, C57BL/6J APCmin mice model	HCT15: 7.53 ± 0.15 μM HT29: 9.05 ± 1.65 μM COLO205: 8.36 ± 0.04 μM HCT116: 8.75 ± 1.58 μM SW480: 27.72 ± 10.96 μM SW620: 22.39 ± 15.17 μM	Glycolysis	GLUT1	Downregulated GLUT1 and activated AMPK to inhibit glycolysis, and inhibited the phosphorylation of p-mTOR, p-P70S6K, and p-4EBP1	[15]
Steroidal saponins	Diosgenin	*Dioscorea opposita* Thunb.	SW1116, RKO, xenograft mouse model	SW1116: 21.15 ± 0.43 μM RKO: 24.06 ± 1.37 μM NCM460: 69.76 ± 1.28 μM	Glycolysis	GLUT2,3,4, PC	Disturbed the aerobic glycolysis and reduce ATP generation	[40]
Steroidal saponins	Dioscin	*Dioscorea opposita* Thunb.	HT29, HCT116, SW620, xenograft mouse model	-	Glycolysis	HK2	Induced Cdh1-mediated Skp2 degradation, thereby inhibiting HK2 expression and glycolysis	[64]
Steroidal saponins	Dioscin	*Dioscorea opposita* Thunb.	HCT116, HT29	-	Glycolysis	HK2	Inhibited glycolysis by restraining HK2, which relates to FBW-7-mediated c-myc degradation	[65]
Alkaloids	Matrine	*Sophora flavescens* Aiton	HCT116, SW620, xenograft mouse model	HCT116: 6.1 μM SW620: 4.9 μM	Glycolysis	GLUT1, HK2, LDHA	Inhibited expression of HIF-1α and its downstream targets to downregulate glycolysis	[47]
Alkaloids	Oxymatrine	*Sophora flavescens* Aiton	HT29, HCT116, hepatic metastasis mouse model of colorectal cancer	-	Glycolysis	PKM2, GLUT1	Double inhibition of PKM2 and downregulation of GLUT1 expression to block aerobic glycolysis	[48]
Alkaloids	Berberine	*Coptis chinensis* Franch.	HCT116	63.6 ± 3 μM	Glycolysis	PKM2	Inhibited PKM2 enzyme activity	[57]
Alkaloids	Lycorine	*Lycoris radiata* (L’Hér.) Herb.	HCT116, HT29, xenograft mouse model	-	TCA cycle	IDH1	Promoted the acetylation of IDH1 to drive the imbalance of mitochondrial dynamics	[77]
Alkaloid derivatives	Halofuginone	*Dichroa febrifuga* Lour.	SW480, HCT116, xenograft mouse model	SW480: 24.83 nM HCT116: 5.82 nM	PPP	G6PD	Inhibited the activity of G6PD and reduced the level of NADPH by regulating Akt/mTORC1 signaling pathway, thereby inhibiting glucose uptake and glycolysis in CRC cells	[101]
Quinones	Curcumin	*Curcuma longa* L.	HCT116, HT29	-	Glycolysis	HK2	Inhibited glycolysis and induces mitochondrial-mediated apoptosis through the regulation of HK2	[24]
Polyphenols	Tannic acid	*Rhus chinensis* Mill.	HCT116, DLD1	DLD1: 53.6 μM HCT116: 43.1 μM FHC: >100 μM	Glycolysis	PKM2	Selectively inhibited the pyruvate kinase activity of PKM2	[55]
Non-flavonoid phenolic compounds	Resveratrol	*Polygonum cuspidatum* Siebold & Zucc.	DLD1	DLD1: 75 ± 4.54 μM	Glycolysis	PKM2	Inhibited PKM2 expression by upregulating miR-326	[62]
Non-flavonoid phenolic compounds	Resveratrol	*Polygonum cuspidatum* Siebold & Zucc.	HCT116, Caco2	HCT116: 50 μM Caco2: 131 μM	Glycolysis	PK	Decreased glucose consumption and the expression of glycolytic enzymes to induce cell apoptosis	[63]
Non-flavonoid phenolic compounds	Resveratrol	*Polygonum cuspidatum* Siebold & Zucc.	Caco2, HCT116	-	TCA cycle	PDH	Targeted the PDH complex, leading to enhanced PDH activity	[86]
Non-flavonoid phenolic compounds	Resveratrol	*Polygonum cuspidatum* Siebold & Zucc.	HT29	-	PPP	G6PD, TKT, PGD	Regulated TKT, G6PD to inhibit pentose phosphate pathway	[100]
Polyphenols	Epicatechin gallate(ECG)	*Acacia catechu* (L. f.) Willd.	HT29	-	PPP, fatty acid metabolism	TKT, G6PD	Regulated de novo synthesis of fatty acids and the PPP way. Inhibited key enzymes	[99]
Sesquiterpenes	Parthenolide derivatives	*Tanacetum parthenium* (L.)	HT29, SW480, HCT116	HT29: 0.66 μM SW480: 0.22 μM HCT116: 1 μM NCM460: 2 μM	Glycolysis	PKM2	Impeded the nuclear translocation of PKM2, fostering a metabolic shift from aerobic glycolysis to oxidative phosphorylation	[50]
Sesquiterpene lactones	Xanthatin	*Xanthium* strumarium L.	HT29, HCT116	-	Glycolysis	GLUT1, MCT4	Reduced Glut1 and MCT4 mRNA and protein levels by inhibiting the phosphorylation of mTOR, 4E-binding protein 1 (4E-BP1) and c-myc	[78]
Sesquiterpene lactones	Hemistepsin A e	*Hemisteptia lyrata* (Bunge) Fisch. & C.A.Mey.	DLD1, CT26, murine allograft model	DLD1: 10.31 ± 0.5536 μM CT26: 9.27 ± 1.497 μM Detroit 551: 52.72 ± 9.042 μM	TCA cycle	PDK1	Inhibited PDK1 activity and decreased lactate production, thus promoting and switching metabolic patterns from glycolysis to OXPHOS	[93]
Diterpenes	Oridonin	*Isodon rubescens* (Hemsl.)	HCT15, COLO205, HCT116, RKO, SW480, SW620, xenograft mouse model	HCT15: 14.105 μM COLO205: 10.272 μM HCT116: 32.977 μM RKO:20.552 μM SW480: 13.373 μM SW620: 11.774 μM	Glycolysis	GLUT1, MCT1	Altered energy homeostasis in cancer cells through downregulating GLUT1 and MCT1 by inhibiting AMPK	[72]
Triterpenoids	Rhus chinensis triterpenoids extract	*Rhus chinensis* Mill.	SW620, HCT116	SW620: 112.3 μg/mL HCT116: 89.6 μg/ml	Glycolysis	GLUT1, LDHA, PKM2, MCT1	Inhibited the expression of glucose metabolism enzymes to mediate lactate transport, and affect the ASIC2-mediated calcineurin/NFAT1 pathway	[73]
Bicyclic sesquiterpenoids	β-caryophyllene	*yzygium aromaticum* (L.) Merr. & L.M.Perry	CT26, xenograft mouse model	-	Glycolysis	ART1	Inhibited ART1-induced glycolysis through AKT/mTOR pathway	[79]
Quinones	Thymoquinone	*Nigella sativa* L.	HCT116, SW480	HCT116: 21.71 μM SW480: 20.53 μM	Glycolysis	HK2	Inhibited HK2-mediated glycolytic metabolism via the PI3K-AKT/HK2 pathway	[71]
	water extracts	*Myristica fragrans* Houtt.	HT29	31.8 ± 1.8 μg/mL	Glycolysis	LDHA	Downregulated LDHA enzyme activity, thereby decreasing lactate production and glucose uptake	[80]
	Alcoholic extract	*Withania somnifera* (L.) Dunal	Azoxymethane-induced colon cancer animals	-	TCA cycle	ICDH, SDH, MDH,α-KGDH	Increased activities of TCA cycle key enzymes and four electron transport chain complex enzymes	[92]

Colorectal cancer cell: SW480, SW620, HCT116, HT29, HCT15, COLO205, RKO, CT26, DLD1, SW1116, LS174T, and Pt2377. Normal human fibroblast cells: Detroit 551. Normal human colon mucosal epithelial cell: NCM460. IC50 of 5-fluorouracil (5FU): colorectal cancer cell IC50:HCT116 2.5 μg/mL; HT29: 0.23 μM, DLD1: 6.5 μM; SW480: 113.10 μg/mL; SW620: 4 μM; HCT8: 52.30 ± 6.80 μM; HCT15: 9.49 ± 2.43 μg/mL; COLO205: 68.1 ± 4.8 μg/mL; SW1116: >400 μg/Ml; LS174T: 3.436 μM. Normal human colon mucosal epithelial cell NCM460: >100 μg/mL.

### 2.2. Natural Products Regulate Amino Acid Metabolism

Amino acids are one of the main energy supply substances in the body. The energy and substances produced in the metabolic process of synthesis and decomposition of amino acids are necessary to maintain body activity and constitute the body. In addition, amino acid metabolism is one of the three major metabolic processes of the body, and involves glutamine metabolism, serine and glycine metabolism, cysteine metabolism, and other pathways [104]. In addition to meeting the energy requirements, tumor cells have more vigorous nutritional requirements than normal cells, because they require more amino acids for biosynthesis. In addition, amino acids can maintain the REDOX state, maintain tumor cell survival, and prevent apoptosis. Studies have shown that abnormal amino acid metabolism plays a crucial role in tumor growth, the tumor microenvironment, and immunity [105].

Glutamine is a conditionally essential amino acid and an important substance for the growth and metabolism of tumor cells, which is required for tumor cells survival in vitro [106]. Glutaminase (GLS) catalyzes the conversion of glutamate and ammonia into other intermediate products to supply nitrogen for other amino acids, nucleic acids and hexosamines. Furthermore, it is metabolized through the TCA cycle to replenish depleted intermediates and facilitate energy production [107] In addition, it is an alternative carbon donator for the synthesis of lipid, and it participates in the synthesis of Glutathione (GSH), a major cellular antioxidant. Glutamine is one of the main energy sources for some tumor cells, and tumor cells consume large amounts of glutamine [108]. Thus, glutamine metabolism or glutaminolysis is equally important for reprogramming CRC cell metabolism by supporting ATP, glutamine metabolism is a promising therapeutic target, and current drug strategies specifically target the inhibition of glutaminase [109].

Epicatechin gallate (ECG) exerted antitumor effects by reducing glucose uptake, increasing glutamine consumption, and decreasing glutamate enrichment, which changes glutamate metabolism [99]. Curcumol has been identified as a potent agent that promotes the degradation of HIF1α, effectively inhibiting the epithelial-mesenchymal transition (EMT) and consequently reducing the invasive and migratory capabilities of CRC cells. Moreover, it impeded the GLS1-mediated metabolism of glutamine, a critical pathway that fuels the proliferation of CRC cells [110].

The MDR in SW620/Ad300 cells is related to the upregulation of spermine synthesis, and D-glutamine metabolism. Curcumin can inhibit spermine and spermidine biosynthesis by decreasing ornithine decarboxylase (ODC) expression, thus decreasing the antioxidative stress and P-gp transport monomer activity of SW620/Ad300 cells [111]. The antitumor mechanism of triptolide may depend on the correction of branched-chain amino acid metabolism disorders, serine/glycine/methionine biosynthesis, and ketone body metabolism disorders [112]. Lobetyolin induces apoptosis and inhibits glutamine metabolism through the ASCT2 signaling pathway in HCT116 cells, leading to a reduction in glutamine-related biomarkers and enhancement of the apoptotic process [113] (Table 2).

### 2.3. Natural Products Regulate Lipid Metabolism

Lipids serve as one of the principal energy sources for the human body and play key roles in both energy and synthetic metabolism for energy supply and storage. Owing to their vast diversity and intricate molecular structures, lipids are important substances that constitute cells and maintain life activities [114]. Lipid metabolism, encompassing the mechanisms of lipid uptake, biosynthesis, and catabolism, is critical for cellular homeostasis. Dysregulation of these processes, manifesting as augmented lipid assimilation, escalated endogenous de novo fatty acid synthesis, intensified fatty acid oxidation, and excessive cholesterol accumulation, has been intimately associated with tumorigenesis. Such metabolic aberrations are known to foster neoplastic growth and advancement [115]. In neoplastic cells facing augmented metabolic demands, surplus lipids and cholesterol are sequestered within lipid droplets. These cells catalyze the de novo biosynthesis of fatty acids (FAs) via enzymatic activation, ensuring an uninterrupted supply requisite for membrane biogenesis, energy generation, and posttranslational protein modifications. Throughout oncogenesis, the fluctuating availability of nutrients necessitates the interplay between oncogenic signaling and lipid metabolic pathways, orchestrating a synergistic modulation that sustains cancer cells’ rapid proliferation, survival, migration, invasion, and metastatic potential. This adaptive metabolic reprogramming is intricately linked to the upregulation of specific signaling cascades, in addition to alterations in pertinent metabolic enzymes and transcription factors [116]. Currently, the molecular constituents of lipid metabolism, including enzymes and transcription factors, are recognized as prospective pharmacological targets in oncology.

FAs constitute the principal components of lipids and are synthesized de novo by an ensemble of enzymes including fatty acid synthase (FASN), ATP-citrate lyase (ACLY), and acetyl-CoA carboxylase (ACC) [117]. Lipid metabolic pathways and related enzymes are potential targets for tumor diagnosis and treatment [118]. ASN, which is the critical target of various natural products that inhibit fatty acid synthesis and CRC progression, plays a crucial role in the synthesis of FAs. Oridonin can effectively inhibit the mRNA and protein expression of FAS and SREBP1 in human CRC cells, subsequently reducing the transcriptional activity of the FAS promoter, which contains the sterol regulatory element binding protein 1 (SREBP1) binding site. This inhibitions reduces cellular fatty acid levels, and increase the apoptosis of CRC cells [119]. RA-XII downregulated the expression of FASN and SCD, thereby inhibiting fatty acid synthesis and reducing fatty acid levels [120]. Berberine downregulated the expression of key lipogenesis enzymes (ACC, ACL, FASN) and inhibited lipid synthesis, which is related to the Wnt/β-catenin pathway [121]. PLA2G16, an adipocyte-specific phospholipase A2, commands the critical regulation of lipid metabolism, and serves as an essential rate-limiting enzyme in the biosynthesis of free fatty acids. Notably, PLA2G16 is implicated in the malignant progression of CRC, predominantly through the suppression of the Hippo signaling conduit—a guardian of cellular growth and organ size. Ginsenoside compound K (GCK), a saponin derived from traditional Chinese medicinal roots such as ginseng and *Panax notoginseng*, has proved that it has good pharmacological activities such as anti-inflammatory, antiatherosclerosis, neuroprotection, and antitumor effects [122,123,124]. GCK exhibited significant inhibitory effects on LOVO cells (Table 3). The anticarcinogenic potential of GCK is intrinsically linked to its inhibitory impact on PLA2G16 protein expression [125].

Fatty acids are dehydrogenated to form unsaturated fatty acids, and polyunsaturated fatty acid derivatives have important physiological functions. Trichothecin (TCN), a sesquiterpenoid found in the endophytic fungus herbaceous *Maytenus hookeri* Loes, reduces the production of unsaturated FAs by blocking SCD-1 activity and impairing tumor cell invasion and metastasis [126]. Short-chain fatty acids (SCFAs) are saturated fatty acids with a chain length of 1–6 carbon atoms that can maintain the integrity of the intestinal wall barrier and prevent intestinal inflammation. EPS-1 is a natural polysaccharide composed of glucose, mannose, galactose, and fructose, that has good antitumor and immune-enhancing effects [127]. EPS-1 changed the composition of the fecal microbiota and increased the concentration of total SCFAs in azomethane/dextran sulfate sodium (AOM/DSS) -induced colorectal cancer mice to inhibit the progression of CRC [128].

Mirabilite, a sulfate class mineral traditionally utilized in clinical settings as Chinese herbal medicine, is recognized for its cathartic and laxative properties. Contemporary research has substantiated its pharmacological activities, including anti-inflammatory effects, immunomodulation, and the facilitation of small intestinal motility [129,130]. Lipidomic studies have revealed that extracts of mirabilite modulate retinol metabolism, propionate metabolism, and glycerophospholipid metabolism, with seven significant biomarkers identified. This evidence suggested that mirabilite extracts may exert anti- CRC effects by influencing lipid metabolism pathways [131].

CRC cells absorb fatty acids from surrounding fat cells and promote oxidative metabolism, consequently providing raw materials for the growth of CRC cells. Increasing fatty acid oxidative breakdown to reduce fatty acid levels in CRC cells is an approach to inhibit CRC progression. Oroxylin A inactivates HIF-1α and modulates fatty acid metabolism, resulting in the downregulation of lipid uptake and synthesis. Moreover, it enhances fatty acid oxidation, consequently reducing intracellular fatty acid levels [132].

The bulb of *Allium cepa* L., commonly known as onion, is replete with an array of phytoconstituents such as volatile oils, phenylpropanoids, flavonoid complexes, and steroidal saponins. Flavonoids constitute a principal bioactive cohort within onions and have multifaceted pharmacological properties, including antineoplastic, and anti-inflammatory effects [133,134]. Related studies have shown that these flavonoid compounds enhance lipid metabolic pathways, substantively decreasing the levels of apolipoprotein B— a critical modifier of low-density lipoprotein (LDL) and total cholesterol (TC)—thereby inhibiting the progression of CRC [135]. In ApcMin/+ mice, kaempferol may reverse the reduction in BA levels by increasing the CYP27A1 and CYP8B1 protein levels in the liver, upregulates the expression of BSEP and promotes hepatic efflux of BA, thereby regulating BA homeostasis, increasing FXR protein expression, and inhibiting Wnt/β-catenin pathway activation [52].

**Table 3:** Natural products regulate lipid metabolism enzymes in CRC.

Medicinal Plant	Bioactive Compounds	Medicinal Plant	Cancer Model	IC50	Metabolic Regulation	Targets	Potential Mechanisms	References
Flavonoids	Oroxylin A	*Scutellaria baicalensis* Georgi	HCT116, Xenograft mouse model	-	Lipid metabolism	HIF1α, FASN, SREBP, CPT	Inactivated HIF1α and regulates fatty acid metabolism, by blocking the Wnt signaling pathway	[132]
Flavonoids	Onion Flavonoids	*Allium cepa* L.	Hyperlipidemia-subcutaneously heterotopic colorectal cancer orthotopic transplant model	-	Cholesterol metabolism	apoB TC	Regulated lipid metabolism, and decreased levels of apoB and TC	[135]
Flavonoids	Kaempferol	*Kaempferia galanga* L.	ApcMin/+ mice	-	BAs metabolism	CYP27A1, CYP8B1	Increased the protein content of liver CYP27A1 and CYP8B1. Upregulated the expression of BSEP to regulate the BA homeostasis, and inhibited the Wnt/β-catenin pathway by increasing the protein expression of FXR.	[52]
Alkaloids	Berberine	*Coptis chinensis* Franch.	DLD-1, Caco-2, Xenograft mouse model	-	Fatty acid synthesis	ACC, ACL, FASN	Downregulated the expression of key enzymes of lipogenesis and inhibited lipid synthesis through the SCAP/SREBP-1 pathway, which is related to the Wnt/β-catenin pathway.	[121]
Saponins	Ginsenoside Compound K(GCK)	*Panax ginseng* C. A. Mey.	SW480, HT29, HCT116, LOVO, Xenograft mouse model	SW480: 46.07 μM HT29: 43.8 μM LOVO: 19.72 μM	Lipid metabolism	PLA2G16	Inhibited the protein expression of PLA2G16 to correct the abnormal lipid metabolism, and regulated the biosynthesis of FFA	[125]
Sesquiterpenes	Trichothecin (TCN)	endophytic fungus of *Maytenus hookeri* Loes	HCT116, LOVO, Xenograft mouse model	-	monounsaturated FA (MUFA) biosynthesis	SCD-1, SREBP1, FAs	Reduced production of unsaturated FAs by blocking SCD-1 activity	[126]
Diterpenoids	Oridonin	*Isodon rubescens* (Hemsl.) H.Hara	SW480, SW620	SW480: 20.79 μM SW620: 37.02 μM	Fatty acid synthesis	FAS	Inhibited the mRNA and protein expression of FAS and SREBP1 and reduced the level of cellular fatty acids	[119]
Glycosides	RA-XII	*Rubia yunnanensis* Diels	HCT116	-	Fatty acid synthesis	SREBP1, FASN, SCD	Reduced fatty acid levels by decreasing the expression of SREBP1 and inhibiting the expressions of de novo fatty acid synthesis proteins FASN and SCD	[120]
Polysaccharides	EPS1-1	*Rhizopus stolonifer* (Ehrenb.) Vuill.	AOM/DSS-induced mice	-	Lipid metabolism	SCFAs	Modulated gut microbiota, increased the concentration of total SCFAs	[128]
	Mirabilite extract	Mirabilite	APCmin/+ mice model	-	Lipid metabolism		Mirabilite influences six lipid metabolic pathways	[131]

### 2.4. Natural Products Regulate Nucleotide Metabolism

Nucleotides include purine nucleotides and pyrimidine nucleotides, which are essential raw materials for the synthesis of DNA and RNA. Nucleotides participate in metabolism and physiological regulation and play crucial roles in vital biological processes [136]. Nucleotide metabolism includes anabolism and catabolism. In tumor cells, enhanced nucleotide synthesis is induced to satisfy the energy requirement [137]. Anabolism involving de novo synthesis and salvage pathways. De novo synthesis utilizes newly synthesized nitrogen-containing heterocycles derived from amino acids, one-carbon units, CO_2_, and other sources. The salvage pathway utilizes free nucleobases within the cell. Notably, substrates for nucleotide biosynthesis are derived from glycolysis, the PPP, the serine-glycine pathway, the TCA cycle, and glutamine transaminase reactions. Affecting other metabolic pathways often results in simultaneous changes in nucleotide metabolism.

Hydroxycamptothecin (SN38) is a natural plant extract isolated from *Camptotheca acuminata* Decne, which has a cell viability inhibitory effect comparable to that of 5-FU (Table 4). SN38 induces DNA damage, further disrupting ribonucleotide (RNP) and deoxyribonucleotide (dRN) metabolism to change the synthesis of DNA and RNA. Moreover, SN38 regulates the expression of ribonucleotide reductase (RNR) to change the levels of ATP, UTP, dATP, and TTP, which are identified as critical metabolites of nucleotide metabolism [138]. A dimeric metabolite of 3,3′-diindolylmethane (DIM), has been obtained from cruciferous vegetables. DIM inhibited the expression of pyrimidine synthesis-related genes (CAD, DHODH, UMPS, NME1, RNR, and CTPS), and increased the expression of the pyrimidine-catabolism-associated gene UPP1. In addition, DIM decreases the total contents of UTP and CTP, as well as blocks DHODH to disrupt pyrimidine synthesis, thus inhibiting the malignant progression of CRC [139]. Rabdosianone I is a bitter diterpene from the *herbIsodon japonicus* (Burm. f.) H. Hara, which inhibits the mRNA and protein expression of TS by directly binding to the mitochondrial inner proteins ANT2 and PHB2, thereby reducing the proliferation of CRC cells [140].

### 2.5. Natural Products Regulate Multiple Metabolism

Natural products regulate a variety of metabolic pathways, with multitarget and multipathway characteristics. Peiminine is an isosteroidal alkaloid derived from a variety of plants, such as *Fritillaria Liliaceae*. Peiminine regulates oxidative stress to change carbohydrate, amino acid and lipid metabolism in CRC cells by activating the PI3K/AKT/mTOR pathway, thereby inducing apoptosis and autophagy in cancer cells [141]. TER induces the apoptosis of CRC cells and affect eight key points, namely, ENO1, ALDOA, PFKFB3, PKM2, and LDHA, thereby exerting anti-CRC effects through key glycolysis and glutamine hydrolysis pathways [142]. Morin and esculetin inhibit aerobic glycolysis and glutaminolysis in colon cancer cells promoted by c-Myc through the suppression of β-catenin [143]. Flexibilide, isolated from the soft coral *Sinularia flexibilis* Quoy & Gaimard, regulates sphingolipid, alanine, aspartate, glutamate metabolism, d-glutamine and d-glutamate metabolism in HCT116 cells. Flexibility impedes the biosynthesis of sphingolipids and glycerophospholipids in phosphatidylcholine (PC) metabolism. This inhibition may be attributable to the blockage of the TCA cycle, concurrent with the activation of TNF receptor-associated factor 2 (TRAF2) and caspase-8, culminating in apoptosis [144].

The chemopreventive effect of *Panax quinquefolius* L. was associated with the alleviation of impaired amino acid, carbohydrate, and lipid metabolism. Arachidonic acid, linoleic acid, glutamic acid, docosahexaenoic acid, tryptophan, and fructose were significantly altered by American ginseng, all of which are associated with inflammation and oxidation [145]. Another study showed that the levels of 28 metabolites related to tryptophan metabolism, glycerophosphatidylcholine metabolism, and unsaturated fatty acid biosynthesis were affected in APCmin/+ mice, and mirabite protected against CRC mainly by regulating tryptophan metabolism and glycerophosphatidylcholine metabolism [146]. Evodiamine (EVO), an indole alkaloid extracted from the plant *Evodia rutaecarpa* (Juss.) Benth., confers a protective effect against AOM/DSS-induced CRC in mice, promoting the enrichment of SCFA-producing bacteria, and facilitating an alteration in microbial metabolism, with a particular emphasis on tryptophan metabolism [147]. Honokiol (HNK), an active polyphenolic compound, was extracted from *Houpoea officinalis* (Rehder & E. H. Wilson) N. H. Xia & C. Y. Wu. In APCmin/+ mice, metabolomics results showed that HNK regulates tryptophan metabolism, the TCA cycle, and the PPP [148].

## 3. Natural Products Regulate Metabolism Related Genes and Pathways

### 3.1. p53

**Figure 2:**
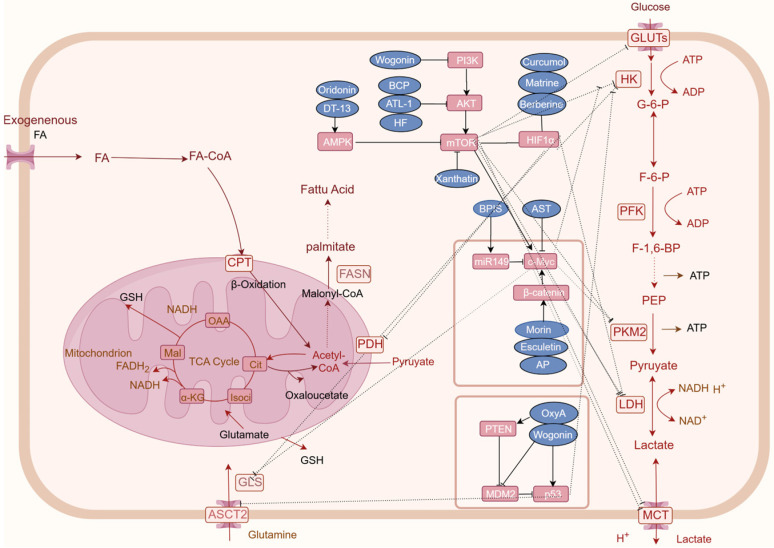
**Natural products regulating key factors and signaling pathways that are involved in CRC cancer metabolism reprogramming.** Natural products may regulate oncogenes genes and related signaling pathways to regulate tumor cell metabolism, such as HIF-1α, MYC, and p53, PI3K-AKT-mTOR, and AMPK pathway. Rectangular boxes indicate altered glucose metabolism enzymes and targets; Blue boxes represent natural products; arrows represent metabolic directions (created on www.figdraw.com).

The p53 gene, an important tumor suppressor, is the most frequently mutated gene in human cancers and often undergoes functional impairment during the progression of CRC [149]. Dysregulation of p53 triggers a cascade of metabolic disruptions, constituting a critical mechanism in CRC progression. Oroxin A, a flavonoid derivative isolated from *Scutellaria baicalensis* Georgi., exerts its anticancer effects on CRC by mediating aerobic glycolysis in a p53-dependent manner both in vivo and in vitro. Investigations into oroxin A have revealed its potential to inhibit CRC progression by repressing Sirt3-regulated MDM2 transcription, thereby preventing MDM2-mediated p53 degradation and consequentially diminishing aerobic glycolysis [150]. Furthermore, wogonin induces p53 phosphorylation at Ser15 and Ser20 and acetylation at Lys382, inhibiting MDM2 expression and enhancing p53 stability, to upregulate the expression of p53 and inhibit glycolysis, while concurrently downregulating the mRNA and protein expression of GLUT1, PGM, HK2, GLUT1, PDHK1, and LDHA [38]. Additionally, p53 moulates the impact of lobetyolin on glutamine metabolism in HCT116 CRC cells by influencing ASCT2 transcription and protein expression, thereby regulating apoptosis [113] (Figure 2).

### 3.2. c-Myc

MYC is a proto-oncogene encoding a protein that plays a crucial role in cell growth, differentiation, and apoptosis. As a transcription factor, c-Myc regulates gene expression, influencing cell cycle progression and metabolism. Dysregulation of c-Myc, through overexpression or mutation, promotes tumorigenesis by enhancing anabolic metabolism. This aberrant upregulation of synthetic metabolic pathways drives tumor growth [151]. Astragaloside IV (AST), a principal active component of the traditional Chinese medicinal herb *Astragalus membranaceus* (Fisch.) Bge. var. *mongholicus* (Bge.) Hsiao. AST has been demonstrated to reduce the mRNA expression levels of c-Myc and glycolytic enzymes (LDH-A, Glut-1, and HK2) in a DSS-induced mouse model, suggesting its potential for combating tumor-associated inflammation and maintaining normal glucose homeostasis [152]. Furthermore, compounds such as morin and esculetin impede aerobic glycolysis and glutaminolysis in colon cancer cells promoted by c-Myc, via the inhibition of β-catenin [143]. Bound Polyphenols compounds (BPIS) inhibits aerobic glycolysis mediated by PKM2 through the upregulation of miR-149 expression, which directly targets the 3’-UTR of c-Myc, thereby suppressing the proliferation of HT-29 cells [153]. AP impedes cellular glycolysis in colorectal cancer cells by obstructing the β-catenin/c-Myc/PTBP1 signaling pathway, thereby inhibiting the activity and expression of tumor-specific PKM2 [58] (Figure 2).

### 3.3. HIF1α

HIF1α is one of the key regulatory factors for cell cycle adaptation in hypoxia. In the tumor microenvironment, it becomes activated and simultaneously functions as a modulator of genes associated with glucose transport proteins, glycolytic enzymes, and vascular endothelial growth factors. Such regulation can lead to metabolic reprogramming, indirectly fostering cancer progression [154]. HIF1α is known to activate the expression of target genes involved in glycolysis, which impacts CRC cell growth, proliferation, cell cycle progression, and the Warburg effect. Notably, matrine, downregulates HIF1α-mediated expression of downstream glycolytic targets such as GLUT1, HK2, and LDHA, suggesting its potential to suppress CRC cell growth by inhibiting the Warburg effect [47]. Worenine, an alkaloid from *Coptis chinensis* Franch., targets HIF1α to decrease glycolytic activity and downregulate the expression of glycolytic enzymes (PFK-L, HK2, and PKM2), thereby inhibiting colorectal cancer cell growth, proliferation, and cell cycle progression [155]. In addition to its potential to curtail glucose uptake in colon cancer cells and attenuate the transcription of glucose metabolism-related genes (GLUT1, LDHA, and HK2), berberine be identified its potential by suppressing mTOR-dependent HIF1α protein synthesis, thus inhibiting heightened glucose metabolism in colorectal cancer cells [156]. Curcumin promotes the degradation of HIF1α, inhibits EMT, curtails CRC cell invasion and migration, and suppresses GLS1-mediated glutamine breakdown [110] (Figure 2).

### 3.4. PI3K/AKT/mTOR Signaling Pathway

Phosphatidylinositol 3-kinases (PI3Ks), which include three classes of lipid kinases, are a family of signal transduction enzymes. PI3Ks plays a key role in activating serine/threonine kinase (AKT) within its downstream signaling pathways, which regulates a number of cellular functions including metabolism, growth, proliferation, survival, transcription, and protein synthesis [157]. AKT, also known as protein kinase B (PKB), is overexpressed in a variety of cancers, including CRC, where it plays a critical role in numerous cellular processes such as glucose metabolism, apoptosis, cell proliferation, transcription, and cell migration. AKT modulates cellular metabolism and tumorigenic metabolic reprogramming by phosphorylating an array of downstream targets, thus facilitating tumor proliferation [158]. The phosphorylation of AKT catalyzes the activation of the mechanistic target of rapamycin complex 1 (mTORC1), underscoring the significant role of the PI3K/AKT/mTOR signaling pathway in the metabolic reprogramming of colorectal cancer [159] (Figure 2).

Atractylenolide I (ATL-1) is one of the principal active constituents of *Atractylodes macrocephala* Koidz [16,160]. ATL-1 impeded tumor proliferation by modulating glycolysis, stemness maintenance, and cellular apoptosis through its effects on the AKT/mTOR signaling pathway [16]. Wogonin restricted the growth of xenograft tumors in nude mice by downregulating HIF-1α expression and glycolysis via inhibition of the PI3K/Akt pathway, concurrently suppressing the expression of HIF-1α, glycolytic proteins, and PI3K/Akt [161]. Xanthatin attenuates the phosphorylation of mTOR, eukaryotic translation initiation factor 4E-binding protein 1 (4E-BP1), and c-Myc in HT29 cells, and its impact on energy metabolism is likely linked to the inhibition of the mTOR signaling pathway [78]. Peiminine induced apoptosis and autophagy in CRC cells by activating PI3K/Akt/mTOR signaling and modulating oxidative stress, subsequently altering carbohydrate, amino acid, and lipid metabolism [141]. β-Caryophyllene inhibits ART1-induced glycolysis and increases ATP and lactate production through the AKT/mTOR pathway [79]. Studies indicate that Halofuginone (HF) downregulates the Akt/mTORC1 signaling pathway, mediating the metabolic regulation of HF through aerobic glycolysis and inducing autophagy [101].

### 3.5. AMP-Activated Protein Kinase (AMPK) Signaling Pathway

AMPK functions not only as a pivotal enzyme in the regulation of energy homeostasis but also as a central modulator of eukaryotic cellular and organismal metabolism, serving as a key protein in multiple signaling pathways [162]. Under conditions of nutrient deprivation, HF inactivates ULK1 via the LKB1/AMPK signaling pathway by downregulating ULK1 at Ser317 and Ser777, thereby suppressing autophagy [163]. DT-13 inhibits the growth of CRC by suppressing GLUT1 expression via activation of the AMPK/mTOR pathway [15]. Oridonin deubiquitinated p-AMPK, causing GLUT1 to be downregulated and autophagy induced in cancer cells [72] (Figure 2).

## 4. Synergistic Effect of Natural Products and Chemotherapy Medicine

Although clinical chemotherapy has demonstrated notable efficacy in the control of CRC, significant resistance to chemotherapeutic drugs severely impedes therapeutic outcomes. The molecular mechanisms underlying drug resistance are intricate and remain to be elucidated. Cancer cells exhibit pronounced alterations in glucose metabolism, lipid metabolism, and glutamine metabolism, which strongly differentiate them from normal cells. Current research indicates that the development of drug resistance is associated with metabolic dysregulation in cancer cells. The metabolic pattern of doxorubicin-resistant human colorectal adenocarcinoma cells (Lovo-DOX) transitions from OXPHOS to the glycolytic pathway, as evidenced by the marked increase in GLUT1 mRNA and protein expression levels. Silybin, the primary bioactive component extracted from the seeds of *milk thistles*, acts as a competitive inhibitor of GLUTs [164]. A study demonstrated that silybin reduces glucose uptake in resistant cells by downregulating GLUT1 protein expression. The combination of doxorubicin and silybin has synergistic effects on Lovo-DOX cells and result in selective cytotoxicity toward resistant cells, confirming the heightened dependency of these cells on glycolysis [102]. In combination with doxorubicin, curcumin micelle-started GLUT1 significantly suppressed the growth of CRC cells and increased the therapeutic effect. The GLUT1-targeted CUR and DOX-loaded combination micelles decreased glucose uptake by blocking the GLUT1 transport protein, thereby helping increase treatment effectiveness [165]. The expression of SLC6A14 (ATB0+), a Na^+^/Cl^−^ coupled amino acid transporter, is upregulated in CRC cells to meet their demand for additional amino acids. Targeting ATB0+ can reduce acid uptake and enhance amino acid deprivation. Oxaliplatin (O) and berbamine (B)-coloaded tryptophan (Trp)-conjugated NPs, can inhibit autophagy and induce apoptosis to reverse OXA resistance [166].

5-FU is one of the most commonly used chemotherapeutic agents for the treatment of CRC and is a critical targeted drug of the nucleotide metabolism thymidylate synthase (TS). However, drug resistance limits the effectiveness of 5-FU. In 5-FU-resistant CRC cells, TS mRNA and protein are overexpressed, which is related to the mechanism of drug resistance. Gambogic acid (GA), *Coptidis Rhizoma* Extract (CRE) and its main active compound berberine reduce TS protein expression either alone or in combination with 5-FU, and CRE and GA significantly reduce the IC50 of 5FU [167,168]. The dry roots of *Rauvolfia vomitoria* Afzel (RVA), exhibit pharmacological effects such as neuroprotective, antitumor, and anti-inflammatory effects [169,170,171]. Comparative metabolomic analysis between the 5-FU treatment group and the high-dose combination therapy group revealed 25 distinct metabolites, that predominantly affect lipid and fatty acid metabolic pathways. These findings suggest that the concomitant use of RVA and 5-FU may inhibit cancer cell proliferation by impacting membrane stability and energy supply in carcinogenic cells. These findings imply that RVA could potentiate the efficacy of the chemotherapeutic drug 5-FU, enhancing its antitumor activity through modulation of lipid metabolism and cellular energy homeostasis [172]. Additionally, quercetin is a flavonoid found in many vegetables and fruits, that has been proven to possess many pharmacological activities, such as antioxidant, antibacterial, antidiabetic, and anticancer effects [173,174,175,176]. Quercetin decreases glucose consumption and lactate acid generation in HCT-15 cells by inhibiting MCT. Cytotoxicity of 5-FU in CRC cells was enhanced, which reduced the viability of CRC cells, and promoting cell apoptosis [103]. Cinnamaldehyde suppressed mRNA expression of BRCA1, TOPO1, ERCC1, and TS, and upregulated OPRT mRNA levels in CRC HT-29 and LoVo cells, to enhance the efficacy of either 5-FU or OXA [177].

Multidrug resistance (MDR) is a phenomenon in which cancer cells develop cross-resistance with different functions and structures to different anticancer medicines. Membrane transport-mediated drug efflux is considered the principal mode of resistance. P-glycoprotein (P-gp), which is encoded by MDR1 gene, is a typical member of the ATP-binding box (ABC) transport protein, which is known to mediate the delivery of chemotherapeutic agents in ATP-dependent fashion [178]. The MDR phenotype of SW620/Ad300 cells was closely related to the upregulation of spermidine and spermine synthesis as well as D-glutamine metabolism. Curcumin inhibited spermidine and spermine biosynthesis by decreasing ornithine decarboxylase (ODC) expression and inhibiting the metabolism of D-glutamine, which in turn reduced the ability of antioxidative stress and the transport activity of P-gp in SW620/Ad300 cells, resulting in the reversal of the MDR [111]. The prophylactic effects of American ginseng are linked to the mitigation of disturbances in amino acid, carbohydrate, and lipid metabolism [145].

## 5. Classification and Role of Natural Products Targeting CRC Metabolic Reprogramming

Natural products, due to their abundant sources and significant bioactivity, have shown promising effects on CRC metabolic reprogramming. This paper discusses natural products derived from plants, minerals, and microorganisms. Plant-derived components include polyphenols, alkaloids, quinones, and terpenoids. Polyphenolic compounds, such as quercetin, apigenin, and kaempferol, are diverse, widely available, and easily obtainable. These compounds are abundant in foods and have demonstrated activity in regulating glucose and lipid metabolism, with a good safety profile [58,103]. However, natural polyphenols face challenges in drug development due to poor solubility and stability [179]. Alkaloids, a large category of complex cyclic compounds containing nitrogen, generally exhibit basic properties. For instance, the bioactive compound berberine has multiple pharmacological activities, including anti-inflammatory and antioxidant effects [180]. Berberine targets PKM2 to regulate glycolysis and interferes with ACC to reduce lipid synthesis, but its poor oral bioavailability limits drug development [57,181]. Steroid saponins, which exhibit good pharmacological activity in this study, target HK2 and GLUT to inhibit glycolysis [15,64], but have high cytotoxicity and low bioavailability. Terpenoids, typically possessing cyclic structures, are the most diverse class of compounds [182]. Terpenoids, which intervene in the activities of multiple enzymes such as PKM2, GLUT, MCT1, GLS1, SCD1, and TS, effectively regulate the four major energy metabolism pathways [50,72,110]. Among them, TCN, derived from endophytic fungi, specifically regulates fatty acid metabolism [126]. Mineral-based components, like mirabilite (sodium sulfate), a traditional Chinese medicine, have been shown to regulate lipid metabolism, but their low solubility and poor safety profile pose challenges for research.

## 6. Conclusions and Prospects

Metabolic reprogramming is a hallmark of CRC. Metabolism is primarily facilitated by metabolic pathways comprising enzymatic reactions, with the activity of these metabolic enzymes playing a crucial role in determining the direction and rate of the metabolic processes. In addition to their canonical role in catalyzing metabolites and producing the nutrients needed, metabolic enzymes are also involved in regulating gene expression, DNA repair, cell apoptosis, and the tumor environment in cancers [183]. Therefore, metabolic enzymes are the main target in the discovery of metabolism-targeted drugs of all time. This review delineates the key metabolic enzymes (HK2, GLUTs, PKM2, LDH, PDH, G6PD) modulated in CRC and provides an overview of the effects of 39 natural compounds derived from three species on metabolic enzymes, related proteins, oncogenes, and the interconnected pathways specific to colorectal carcinogenesis.

Except for rich sources, and well-defined pharmacological activities, natural products have several advantages for metabolic drug development [184,185]. Firstly, natural products, with their multitarget and multipathway characteristics, are ideally suited to addressing the complex metabolic alterations in tumors. The metabolism pathways of glucose, amino acids, lipids, and nucleotides are linked to each other through the common intermediate, the TCA cycle, and oxidative phosphorylation [186]. It is suggested that natural products should not be limited to the metabolism of a single substance but may be achieved through multiple pathways and targets. Curcumin regulates a variety of key enzymes involved in glucose metabolism and amino acid metabolism. Natural products modulate multiple substance metabolism, through directly binding to enzymes, changing the expression of genetic material, influencing the activity of enzymes, changing epigenetics, and influencing upstream or downstream regulators [187,188], therefore inhibiting cellular proliferation, inducing apoptosis, activating antitumoral immune responses, and modulating the tumor microenvironment. Secondly, the abundant variety and diverse scaffolds of natural products make them ideal candidates for the screening and development of drugs targeting tumor metabolism. Moreover, natural products, when used in combination with other drugs, exhibit significant synergistic and anti-resistance effects.

However, there are still challenges that hinder the development of metabolic inhibitors derived from natural products. Natural products often suffer from instability, low bioavailability, poor solubility, and suboptimal selectivity, limiting their therapeutic application in oncology [189]. The plasticity of metabolic pathways and the heterogeneity of tumors should be taken into consideration, which complicates the assurance of drug safety. Most natural products cannot precisely target tumor metabolic changes and may affect the metabolism of normal cells. In addition, some natural products exhibit hepatotoxicity and nephrotoxicity. To overcome these challenges, a series of actions must be taken. Integrating multiomics, single-cell, and spatial detection technologies to identify the precise mechanisms of CRC metabolic reprogramming that differ from normal cells is of paramount importance. Further, drug delivery systems, novel materials, and structural modification offer solutions to limitations, thereby expanding the utility of natural compounds [190]. Taken together, development and modification of natural products to target the metabolism of malignant cells are a novel and promising field of drug discovery for the treatment of CRC.

## Figures and Tables

**Table 2:** Natural products regulate amino acid metabolism enzymes in CRC.

Medicinal Plant	Bioactive Compounds	Medicinal Plant	Cancer Model	IC50	Metabolic Regulation	Targets	Potential Mechanisms	References
Quinones	Curcumin	*Curcuma longa* L.	SW620, Dox-selected P-gp overexpresseSW620/Ad300 cells	-	spermine synthesis and D-glutamine metabolism	ODC	Inhibited the biosynthesis of spermidine by decreasing the expression of ODC and suppressed D-glutamine metabolism, thereby reducing the antioxidative stress ability and P-gp transport activity	[111]
Sesquiterpenes	Curcumol	*Curcuma longa* L.	patient-derived orthotopic xenograft (PDOX) CRC mouse model	-	Glutamine metabolism	GLS1, HIF1A	Inhibited GLS1-mediated glutaminolysis, related to stimulate HIF-1α degradation	[110]
Diterpenoids	Triptolide	*Tripterygium wilfordii* Hook. f.	Xenograft mouse model	-	Amid acid metabolism		Regulated branched-chain amino acid metabolism, serine/glycine/methionine biosynthesis, and ketone body metabolism to inhibit CRC	[112]
Polyacetylenes	Lobetyolin	*Codonopsis pilosula* (Franch.) Nannf.	HCT116, Xenograft mouse model	-	Glutamine metabolism	ASCT2	Induced apoptosis and inhibited glutamine metabolism through the ASCT2 signaling pathway.	[113]

**Table 4:** Natural products regulate nucleotide metabolism enzymes in CRC.

Medicinal Plant	Bioactive Compounds	Medicinal Plant	Cancer Model	IC50	Metabolic Regulation	Targets	Potential Mechanisms	References
Alkaloids	Hydroxycamptothecin	*Camptotheca acuminata* Decne.	HCT116	2.33 ± 0.14 μM	synthesis of DNA and RNA	RNR	Regulated the expression of RNR, to disturb RNs and dRNs metabolism, thus changing the synthesis of DNA and RNA.	[138]
Alkaloids	3,3’-Diindolylmethane (DIM)	Cruciferous vegetables	DLD1, HCT116	-	Pyrimidine synthesis and catabolism	CAD, DHODH, UMPS, NME1, RNR, CTPS, UPP1	Inhibited the expression of CAD, DHODH, UMPS, NME1, RNR, and CTPS, while increasing expression of UPP1, thereby decreasing the total contents of UTP and CTP, and blocking DHODH to disrupt pyrimidine synthesis	[139]
Diterpenoids	Rabdosianone I	*Isodon japonicus* (Burm. f.) H. Hara	HT29, HCT116	-	DNA synthesis	TS, ANT2, PHB2	Targeted two mitochondrial inner proteins ANT2 and PHB2, and reduced mRNA and protein of TS	[140]

## Data Availability

The main figure were assembled in Figdraw https://www.figdraw.com/ (accessed on 4 September 2024).
